# Fronto-striatal structures related with model-based control as an endophenotype for obsessive–compulsive disorder

**DOI:** 10.1038/s41598-021-91179-2

**Published:** 2021-06-07

**Authors:** Meltem I. Kasal, Lutfullah Besiroglu, Nabi Zorlu, Nur Dikmeer, Aslıhan Bilge, Ercan Durmaz, Serap Polat, Fazil Gelal, Michael Rapp, Andreas Heinz, Miriam Sebold

**Affiliations:** 1 Department of Psychiatry, Ataturk Education and Research Hospital, Katip Celebi University, Izmir, Turkey; 2 Department of Radiodiagnostics, Ataturk Education and Research Hospital, Katip Celebi University, Izmir, Turkey; 3 Department for Social and Preventive Medicine, University of Potsdam, Potsdam, Germany; 4 Department of Psychiatry and Psychotherapy, Charité Campus Mitte (CCM), Charité-Universitätsmedizin Berlin, Berlin, Germany

**Keywords:** Cognitive neuroscience, Obsessive compulsive disorder

## Abstract

Recent theories suggest a shift from model-based goal-directed to model-free habitual decision-making in obsessive–compulsive disorder (OCD). However, it is yet unclear, whether this shift in the decision process is heritable. We investigated 32 patients with OCD, 27 unaffected siblings (SIBs) and 31 healthy controls (HCs) using the two-step task. We computed behavioral and reaction time analyses and fitted a computational model to assess the balance between model-based and model-free control. 80 subjects also underwent structural imaging. We observed a significant ordered effect for the shift towards model-free control in the direction OCD > SIB > HC in our computational parameter of interest. However less directed analyses revealed no shift towards model-free control in OCDs. Nonetheless, we found evidence for reduced model-based control in OCDs compared to HCs and SIBs via 2nd stage reaction time analyses. In this measure SIBs also showed higher levels of model-based control than HCs. Across all subjects these effects were associated with the surface area of the left medial/right dorsolateral prefrontal cortex. Moreover, correlations between bilateral putamen/right caudate volumes and these effects varied as a function of group: they were negative in SIBs and OCDs, but positive in HCs. Associations between fronto-striatal regions and model-based reaction time effects point to a potential endophenotype for OCD.

## Introduction

Recent theories suggested that an imbalance between goal-directed and habitual control—two distinct and also parallel interacting decision-making strategies—may be a potential pathophysiology underlying obsessive–compulsive disorder (OCD)^1^. From a computational perspective, goal-directed and habitual control arise from two different reinforcement learning strategies, known as model-based and model-free learning, respectively^2^. Model-based (MB) control is prospective and relies on predicted consequences of actions. Although MB control can be computationally costly because it requires the explicit consideration of future outcomes, it can flexibly adapt behavior following sudden environmental changes. In contrast, model-free (MF) control is computationally inexpensive and fast but at the cost of flexibility.

A prominent task to assess MF and MB is the two-step task^3^, as it allows to distinguish differential contributions of both control systems. More precisely, this task builds on the assumption that MF choices lack the incorporation of a mental model of the precise task structure whereas MB choices do consider this structure. By using this task, two previous studies have shown a significant shift towards MF control in patients with OCD compared to healthy controls^4,5^. Furthermore, a subseqent study in a general population sample reported a negative correlation between self-reported OCD symptoms and MB control^6^. Interestingly, in this study not only OCD symptoms but also other symptoms that scored on a factor of compulsivity (e.g. addiction, eating disorder) predicted reductions in MB control. Thus, imbalance between MB to MF control has been suggested to serve as a transdiagnostic marker for disorders of compulsivity. However, it is yet unclear, whether this shift in the decision process is also heritable. First-degree relatives of OCD patients are particularly at greater risk for developing the disorder^7^, suggesting a familial component to the disorder. Endophenotypes are cognitive traits that are illness related but also observed in clinically unaffected family members. Importantly such traits might point to disorder relevant neurobiological substrates and mechanisms^8^. To our knowledge, no study has yet examined whether the balance between MF and MB serves as an endophenotype for OCD.

Regarding the neurobiological basis of the balance between MF and MB control, previous studies in healthy subjects suggested an important role of the medial and lateral prefrontal cortex and striatum^5,9,10^. These brain regions also broadly overlap with the model implicating abnormal frontostriatal circuits underlying OCD^11,12^. However, to date there is limited evidence that these dysfunctional fronto-striatal circuits are associated with a shift away from MB control, that is prevalent in OCD^13^.

In this study, we applied a two-step task⁠ to examine possible differences in the balance between MB and MF control in patients with OCD and unaffected siblings, relative to healthy controls. First, we hypothesized that behavior in OCD patients would shift towards MF control relative to healthy controls. Further we assumed, that siblings would have intermediate levels in this measure. We investigated these hypotheses via applying a computational model and analyses of choice and reaction time (RT) data. Last, we tested the idea, that prespecified brain structures (prefrontal cortex and striatum), related with the balance between MF and MB control served as an endophenoytpe for OCD.

## Results

### Participants characteristics

The groups were matched for age, gender, education level and pack years of cigarette smoking. Table 1 shows the demographics and clinical data.

**Table 1:** Participant sociodemographic and clinical characteristics.

	OCD (n = 32)	SIB (n = 27)	HC (n = 31)	Statistics and post-hoc effects of group
Age	32.2 ± 9.8	33.5 ± 11.0	32.0 ± 9.7	F = 0.196, *p* = 0.822
Education (years)	12.3 ± 3.8	11.6 ± 4.0	13.0 ± 4.6	F = 0.789, *p* = 0.558
Sex (male/female)	15/17	11/16	13/18	X^2^ = 0.262, *p* = 0.877
Pack years of smoking	3.5 ± 5.9	4.6 ± 8.1	5.7 ± 8.2	X^2^ = 1.295, *p* = 0.523
Cognitive speed (DSST)	42.1 ± 16.8	50.2 ± 15.6	57.7 ± 16.0	F = 7.353, *p* = 0.001 HC > OCD (*p* = 0.001)
Verbal working memory (DS)	5.6 ± 2.2	5.3 ± 2.2	6.7 ± 2.5	F = 4.913, *p* = 0.042 HC > OCD (*p* = 0.048) HC > SIB (*p* = 0.020)
OCD age of onset	25.0 ± 9.5			
**DYBOCS severity**				
Aggressiveness	6.5 ± 4.6			
Sex/Religion	4.0 ± 4.7			
Symmetry/Ordering	5.1 ± 4.9			
Contamination/Cleaning	6.1 ± 4.8			
Hoarding	1.7 ± 3.5			
Miscellaneous	3.3 ± 4.4			
Total	18.0 ± 4.8			

Data are presented as mean ± standard deviation.

*HC* healthy controls, *OCD* patients with obsessive–compulsive disorder, *SIB* unaffected siblings, *DYBOCS* dimensional scale for the assessment of the presence and severity of obsessive–compulsive symptoms, *DSST* Digit Symbol Substitution Test, *DS* digit span test.

### Behavioral data

**Figure 1:**
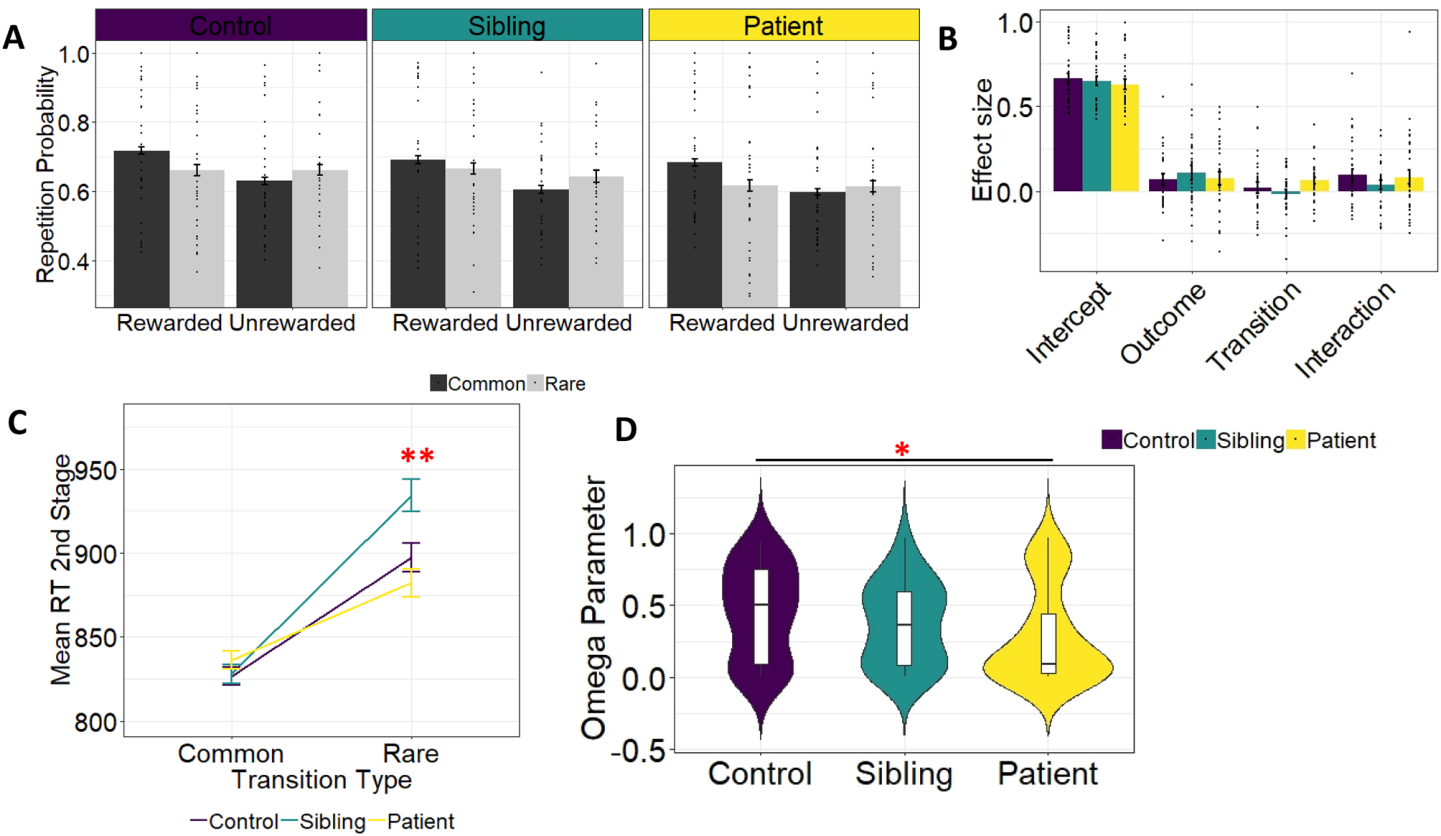
(**A**) Repetition probabilities and (**B**) results of the regression analysis where we regressed stay/switch behavior on outcome (Reward, No Reward), transition (Common, Rare) and group (Control, Sibling, OCD). We found no evidence that groups showed significant different choice behaviors in model-free (Outcome effect in Figure **B**) or model-based (Interaction effect in Figure **B**) control. Moreover, general stay/switch behavior was not significant different between groups (Intercept effect in Figure **B**) and choice behavior was not different across groups after rare or common trials (Transition effect in Figure **B**). (**C**) Mean 2nd stage reaction time as a function of transition type and group (MB RTs). OCDs discriminated less between common and rare trials in their RTs (common: m = 836 ms, sd = 351 ms, rare: m = 882 ms, sd = 361 ms) compared to HCs (common: m = 826 ms, sd = 352 ms, rare: m = 897 ms, sd = 365 ms, group difference, p < 0.01) and SIBs (common: m = 828 ms, sd = 342 ms, rare: m = 934 ms, sd = 385 ms, group difference, p < 0.001) (indicated with **). The difference between SIBs and HCs showed trendwise significance (p = 0.0503). (**D**) Comparison of the computational parameter ω, indicating the balance between model-free and model-based control, between groups. Jonckheere-Terpstra testing showed ordered difference for the ω parameter (indicated with *).

The overall idea of the task is, that MF and MB strategies predict different 1st stage choices depending on the previous trial’s outcome and transition frequency (see method section). To test whether groups significantly differed in MF vs. MB control, we thus regressed 1st stage choices (stay/switch) on the previous trial outcome (Reward/ No Reward), transition frequency (common/ rare) and group (HC, SIB, OCD). Our results indicated a main effect of outcome (*b* = 0.21*, SE* = 0.04*, CI*_*95%*_ [0.14 0.28], *P* < 0.01) and an interaction between outcome and transition (*b* = 0.37*, SE* = 0.07*, CI*_*95%*_ [0.22 0.51]*, P* < 0.001). Thus, subjects showed a mixture between MB and MF learning (Fig. 1A). We found no effect of group on repetition probability (*P* = 0.69), no interaction between group and outcome (*P* = 0.94), no interaction between group and transition (*P* = 0.21) and no interaction between group, transition and outcome (*P* = 0.31, Fig. 1B). Thus, contrary to our hypothesis, groups showed no difference in behavioral MF and MB measures.

### Reaction time analysis

As previous studies^9,14^ have demonstrated additional model-based signatures in 2nd stage RTs, we additionally tested, whether groups showed significant different model-based RT effects. To this end, we regressed 2nd stage RTs on transition frequency (common/ rare) and group (HC, SIB, OCD). Our results indicated a significant main effect of transition (*b* = − 78.7, *SE* = 8.4, *CI*_*95%*_ [− 95.1 − 62.2]*, P* < 0.001). Overall subjects were faster after common (m = 830 ms, sd = 349 ms) compared to rare (m = 903 ms, sd = 371 ms) transitions. Beyond this, we found an interaction between transition and group (*P* < 0.001). Post-hoc analysis indicated, that OCDs discriminated less between common and rare trials in their RTs compared to HCs (*b* = 22.3, *SE* = 8.3, *CI*_*95%*_ [5.9 38.6], *P* < 0.01) and SIBs (*b* = 39.3, *SE* = 8.9, *CI*_*95%*_ [22.0 56.7]*, P* < 0.001). The difference between SIBs and HCs showed trendwise significance (*b* = − 17.1, *SE* = 8.7, *CI*_*95%*_ [− 34.12 0.02], *P* = 0.0503) (Fig. 1C).

### Computational modelling

In order to gain a mechanistic understanding of the underlying decision process, we fitted several computational models to the choice data. As previously^15^, the seven parameter hybrid model, including the weighting parameter ω (indicating the balance between MF and MB control) was the best fitting model (Supplement 1). To mirror our behavioral choice analyses (see above), we tested, whether this weighting parameter ω was significant different between groups. As hypothesized, Jonckheere-Terpstra testing showed ordered difference for ω values, such that OCD < SIB < HC (*P* = 0.027) (Fig. 1D). Post-hoc Wilcoxon rank sum tests demonstrated trendwise significant difference in ω values between OCDs and HCs (*P* = 0.098). In contrast, there were no significant differences between SIBs and HCs (*P* = 0.336), or between OCDs and SIBs (*P* = 0.266). Additional exploratory analyses, where we analyzed age of onset effects, severity effects (DY-BOCS scores), gender effects and medication status are reported in the Supplement 2 to 3.

We found significant differences between groups in ﻿the digit symbol substitution test^16^ (DSST) measuring cognitive speed and the digit span^16^ (DS) backwards test measuring working memory despite matching for education which ﻿could impact our results. We therefore repeated our analysis after regressing out DSST and DS backwards test scores. We also regressed out age which is shown to impact model-based behavior^17^. After regressing out the effect of age, Jonckheere-Terpstra testing remained significant (*P* = 0.016) and post-hoc Wilcoxon rank sum tests demonstrated significant difference in ω values between OCDs and HCs (*P* = 0.035) but no significant differences between OCDs and SIBs (*P* = 0.267) and SIBs and HCs (*P* = 0.314). However, after regressed out the effects of DSST and DS backwards test scores, Jonckheere-Terpstra testing did not remain significant (*P* = 0.103) and post-hoc Wilcoxon rank sum tests demonstrated no significant difference in ω values between OCDs and HCs (*P* = 0.265), OCDs and SIBs (*P* = 0.352) and SIBs and HCs (*P* = 0.654) which are in line with previous studies that showed an association between general cognitive functions and MB behaviour^18,19^. Therefore, our finding of ordered difference for ω values ﻿should be interpreted with caution.

Further exploratory comparisons of the remaining reinforcement and softmax parameters yielded non-significant group effects (Supplemental 4 and Table S1).

### Correlation analysis

In order to test how strong different measures of MB/MF control were related to each other we set up a correlation matrix between different task measures. In line with previous studies^9,20,21^, we found a significant positive correlation between mean 2nd stage RT effects and ω values (﻿Rho = 0.237, P = 0.024), a significant positive correlation between ﻿the interaction term and 2nd stage RT effects (﻿Rho = 0.533, P < 0.001) and a significant correlation between interaction term and ω values (﻿Rho = 0.239, P = 0.026). However, none of these measures were associated with the Outcome term (all P values > 0.05, See Supplement 5).

### Neuroanatomical correlates of task results

#### Correlations with ω values

There were no correlations between ω values and cortical thickness and surface area within all subjects. For further exploratory analysis see Supplement 6.

#### Correlations with outcome and transition interaction

There were no correlations between ﻿estimated coefficients of the outcome and transition interaction and cortical thickness and surface area neither across all subjects nor within groups.

#### Correlations with 2nd stage RT effects

**Figure 2:**
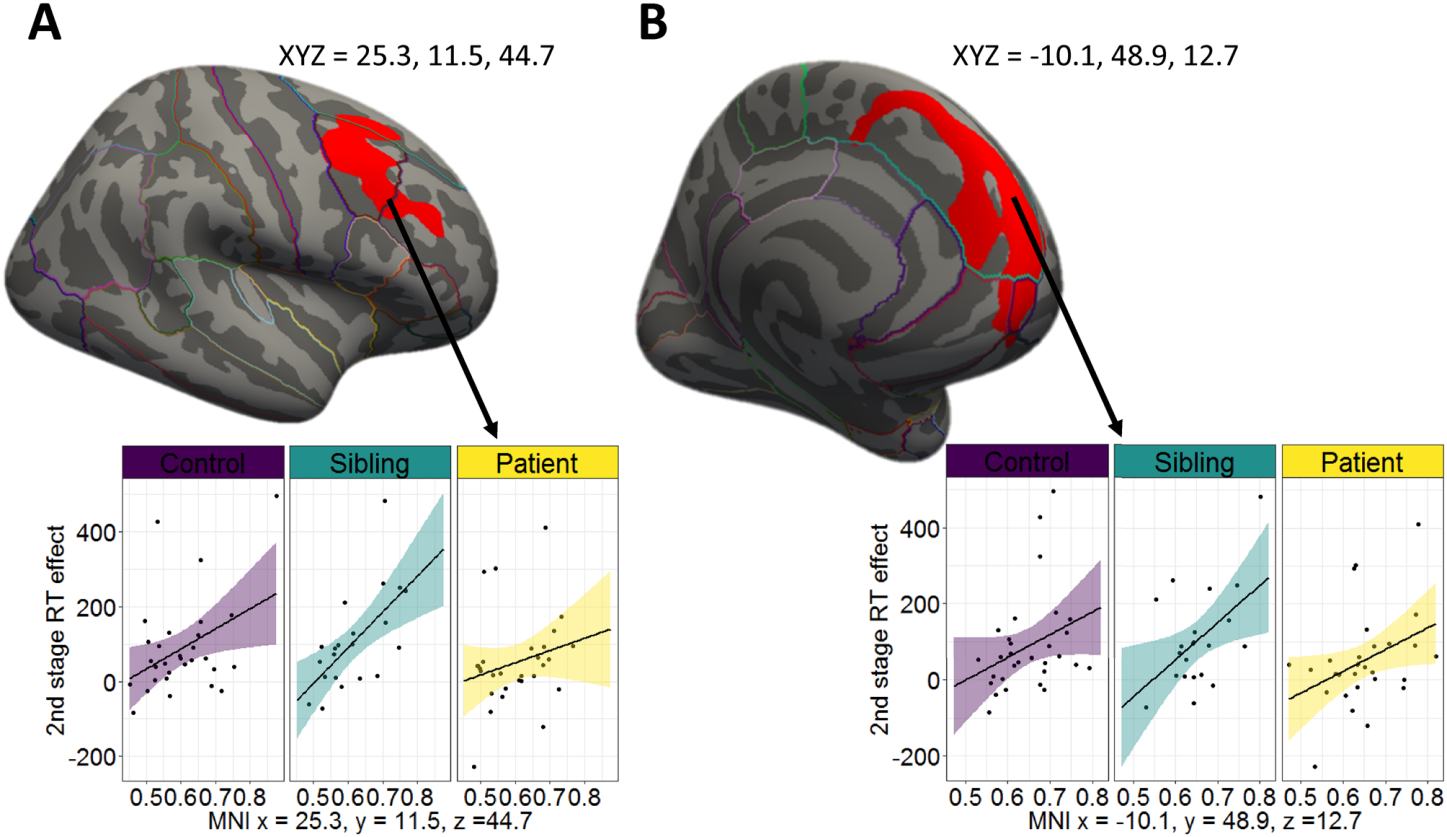
Association between MB-RT effects and surface area across all groups. (**A**) right superior frontal gyrus extending to caudal and rostral middle frontal gyrus (Talairach-coordinates x = 25.3, y = 11.5, z = 44.7, size in mm^2^ = 1928, *cwp* = 0.014) and (**B**) left superior frontal gyrus extending to frontal pole and medial orbitofrontal cortex (Talairach-coordinates x = -10.1, y = 48.9, z = 12.7, size in mm2 = 2043, *cwp* = 0.008).

Within all subjects, there were significant positive correlations between 2nd stage RT effects (mean RT differences for rare versus common states) with surface area values, but not cortical thickness, in two clusters. The first cluster was in right superior frontal gyrus extending to caudal and rostral middle frontal gyrus (*CWP* = 0.014) (Fig. 2A). The second cluster was in the left medial superior frontal gyrus extending to frontal pole and medial orbitofrontal cortex (*CWP* = 0.008) (Fig. 2B). For each cluster, averaged values of surface area were extracted for each individual. Follow-up partial correlation analysis with age and gender as covariates of no interest, showed positive correlations were mainly driven by SIBs rather than HCs and OCDs in first cluster (*r* = 0.604, *P* = 0.006, *r* = 0.361, *P* = 0.060 and *r* = 0.183, *P* = 0.360 respectively) and SIBs rather than HCs and OCDs in second cluster (*r* = 0.481, *P* = 0.037, *r* = 0.312, *P* = 0.105 and *r* = 0.319, *P* = 0.104 respectively).

**Figure 3:**
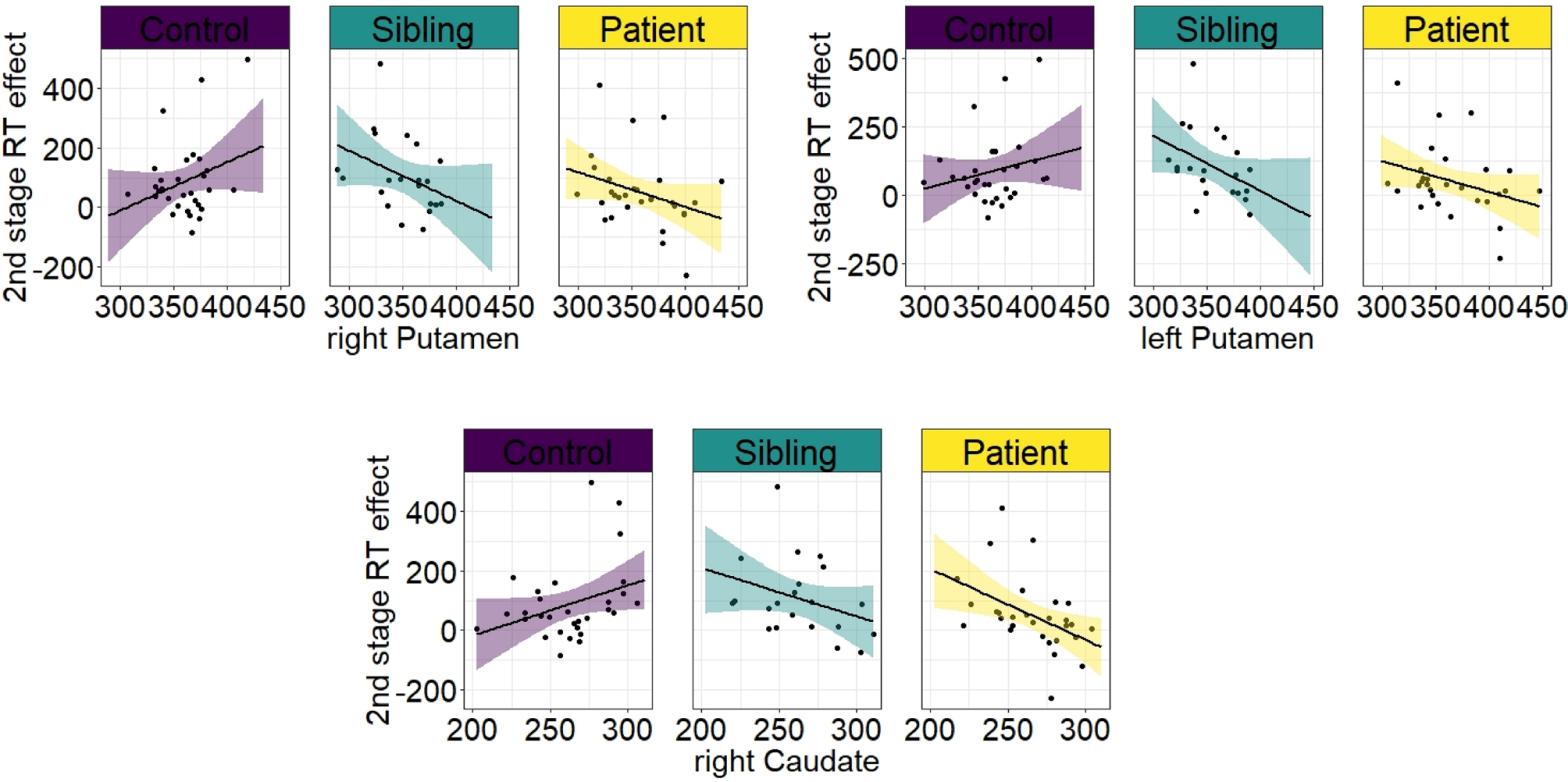
Association between 2nd stage RT effects and normalized volumes (× 10,000) of putamen and caudate nucleus. Across all subjects we found a negative association between 2nd stage RTs and volumes of left and right putamen and caudate nucleus. Interestingly these associations were particularly driven by Siblings and Patients (**A**,**B**) or patients alone (**C**) but not healthy controls (**A**–**C**).

We next examined correlations between 2nd stage RT effects and normalized volumes of putamen and caudate nucleus. For the right putamen, we found a significant negative correlation within all subjects (*Rho* = − 0.291*, P* = *0*.010) which was driven by SIBs and OCDs rather than HCs (*Rho* = − 0.576, *P* = 0.010, *Rho* = − 0.468, *P* = 0.014 and *Rho* = 0.115, *P* = 0.559 respectively) (Fig. 3A). Similiarly, we found a negative correlation for left putamen (*Rho* = − 0.291*, P* = *0*.010) which was mainly driven by SIBs and OCDs rather than HCs (*Rho* = − 0.492, *P* = 0.032, *Rho* = − 0.378, *P* = 0.052 and *Rho* = 0.097, *P* = 0.623 respectively) (Fig. 3B). There was also a negative correlation for the right caudate nucleus (*Rho* = − 0.228*, P* = *0*.045) which was mainly driven by OCDs rather than HCs and SIBs (*Rho* = − 0.550, *P* = 0.003, *Rho* = 0.220, *P* = 0.261 and *Rho* = − 0.280, *P* = 0.246 respectively) (Fig. 3C).

## Discussion

In this study, we used the two-step task in patients with OCD, unaffected siblings of patients with OCD and HCs in order to identify alterations in the balance between model-based and model-free control that may be associated with the familial risk for OCD.

Contrary to our hypothesis, ω values did not significantly differ between OCDs and HCs suggesting no evidence for a shift away from MB control in OCDs. However, albeit not statistically significant OCDs had trendwise lower ω values compared to HCs (p = 0.098). Beyond this, we found significant ordered differences for ω values, in the direction OCD < SIB < HC. All together, these findings suggest that OCD patients showed the expected direction of reduced MB control, although in the present study these deficits seem to be very subtle. The differences between our subtle deficits and more pronounced deficits that have been found in previous studies^4,5^ may be sample related: our study included OCD patients mostly treated with psychotropic medications including antipsychotics and different classes of antidepressants while previous studies included SSRI treated or medication free patients^4,5^. Indeed, different types of drugs have been shown to selectively increase/decrease model-based control^22–24^. Additionaly, given the trendwise significant difference between OCDs and HCs and smaller sample size of our study, particularly in the HC group (n = 31) than previous studies^4,5^ (n = 96 and n = 93 respectively), another possible explanation might be inadequate power. Interestingly, and in line with our null finding regarding the group difference between OCDs and HCs, a very recent study using the same task, suggested that OCD diagnosis per se was not sufficient to explain reductions in model-based control^25^. Instead, in the study by Gillan et al. (2019)^25^, variance in the balance between MB control was best explained by the symptom dimension of compulsivity, which is one clinical phenotype that manifests in OCD but also in other mental health disorders (e.g. addiction). Thus, our null finding might also be due to the fact that OCD is a highly heterogenous disorder with many comorbidities and the deficits in MB control in previous study rather relate to a symptom (compulsivity) than a syndrome.

The primary aim of this study was to test the hypothesis, that the balance between MF and MB control served as an endophenotype for OCD and would therefore be altered in first-degree relatives of OCDs. However, our findings did not support this hypothesis. Similar to our result, one recent study did not find empirical support for reduced MB control as an endophenotype for alcohol dependence^26^. However, we have to note that altough there were no differences between groups, the SIBs showed statistically significant intermediate levels between the OCDs and HCs. We speculate that these intermediate levels might be due to lower expression of high-risk genes in the SIBs compared to the OCDs and/or shared environment effects, but future studies investigating larger sample sizes should test this hypothesis more formally.

Interestingly, although we found no evidence for reduced MB control in OCDs in our behavioral nor computational analyses, we found support for this in our RT analyses. In the two-step task, MB control requires knowledge of the transition structure. Therefore, if a decision is controlled by the MB system any unexpected 2nd stage state should result in slower RTs as opposed to common trials. Indeed, recent studies using the two-step task have included this metric as a variant of MB control^20,27,28^. In line with this, a recent methodological study suggests that the stability of MB estimates in the two-step task could substantially be improved by adding RT data to choice data^20^, which supports the longheld notion of the decision-making literature that RT data is an important source of information^29,30^. Our analyses revealed that OCDs showed less RT differences for rare versus common states compared to SIBs and HCs suggesting decreased transition structure knowledge in the OCDs. However, due to our cross-sectional design, we are unable to establish a clear temporal relationship between higher RT difference for rare versus common states and OCD. A longitudinal study would be required to elucidate these relationships. Critically, the SIBs showed higher RT difference for rare versus common states compared to HCs at a trendwise significance level. However, SIBs could only partly use this transition knowledge prospectively in their subsequent first-stage choices, given the ω values of SIBs were between HCs and OCDs. MB control is computationally costly and previous studies have reported deficits in working memory capacity ﻿attenuates the model-based contributions to behavior^18,19,31,32^. Therefore, reduced working memory capacity observed in DS backwards test that we found in SIBs compared to HCs might be a possible explanation for ﻿inefficient use of transition knowledge in the SIBs. However, we have to note that, although RT effects, ω values and interaction term were related to each other, RT effects do not necessarily have to result in MB choices. For example, RT effect might be more implicit than MB choices and participants might simply ignore the structure of the task despite having knowledge of it.

Given that the 2nd stage RT effects were the most pronounced MB related group effects, we further tested the hypothesis, that the structural correlates explaining variance in this metric would overlap with previous reports on structural correlates in MB control. With regard to this assumption, we found a positive correlation between surface area values of left medial (mPFC) and right dorsolateral prefrontal cortex (dlPFC) and 2nd stage RT effects across all subjects. This is in line with previous studies suggesting a prominent role for these structure in MB control. For instance, previous functional imaging studies found neural signature of MB learning signal in the dlPFC and the intraparietal sulcus in HCs^33,34^. Another study reported impaired MB control after transient disruption of the right dlPFC via theta burst transcranial magnetic stimulation^31^. The mPFC has also been consistently implicated in MB behavior in healthy subjects^3,9,35^. Furthermore, studies in patients with compulsive disorders such as alcohol dependence^15^ and binge eating disorder^36^ have associated blunted activation or reduced gray matter volume of mPFC with reduced goal-directed control. Taken together, our finding of a positive correlation between mPFC/dlPFC cortical surface area and model-based RT effects, might point to a relevant neurobiological substrate of deficient MB control in OCD.

We also found that bilateral putamen and right caudate volumes differentially covaried with 2nd stage RT effects across groups: Whereas we found a negative correlation between bilateral putamen and right caudate volumes and 2nd stage RT differences in SIBs and OCDs, a positive correlation between these volumes and 2nd stage RT differences was found in HCs. These findings are consistent with a number of studies implicating fronto-striatal loops in OCD^37^. For instance, similiar to our findings, a recent resting-state functional imaging study reported reduced functional connectivity between the putamen and the dlPFC, which was associated with altered performance in goal-directed planning in OCDs^38^. Although, there were no performance differences between SIBs and HCs in goal-directed planning in the Tower of London task, Vaghi et al.^39^ also reported hypoactivation of the right dlPFC during goal-directed planning coupled with reduced functional connectivity between dlPFC and putamen in both OCDs and SIBs compared to HCs. In sum, if replicable, our findings augment previous functional imaging studies by suggesting that association between surface area of dlPFC, as well as volume of putamen and model-based RT effects might be a canditate endophenotype.

Limitations should be considered when interpreting the findings of the study. First, cross-sectional nature of the study limits interpretability of our results and longitudinal studies are required to establish causal relationships. Second, our sample was relatively small, particularly in the SIB group, which might reduce the statistical power of the group comparisons and further studies with larger sample sizes are needed to confirm the robustness of our findings. ﻿In addition, symptom severity of OCD and depression were assessed within the patient group only using the Dimensional Yale–Brown Obsessive–Compulsive Scale^40^ and Beck Depression Inventory^41^ respectively, and thus we may have underestimated subclinical symptoms within the SIB and HC groups. Finally, most patients were taking psychotropic medication. Therefore we could not reliably disentangle effects of medication on task results and brain morphology within the OCD group.

In conclusion, OCDs showed less model-based RT effects than HCs and SIBs. In contrast, SIBs showed higher model-based RT effects than HCs at a trendwise significant level suggesting SIBs could only partly use transition knowledge prospectively in their subsequent first-stage choices given the ω values of SIBs were between HCs and OCDs. Association between fronto-striatal regions and model-based RT effects might partly explain behavioral correspondings of fronto-striatal alterations that have been consistently found in OCD.

## Methods

### Participants and screening instruments

A total of 90 participants (32 patients with OCD, 27 siblings (SIB) of patients with OCD and 31 healthy controls (HC)) were enrolled in the study. Exclusion criteria for participants were, current or past history of any serious psychiatric illness, including any psychotic or bipolar disorder; any lifetime substance use disorder (except nicotine); use of psychotropic medication except patients with OCD; any family history of OCD for HC; history of loss of consciousness for more than 30 min; current or past history of any significant neurological disorders; and any severe hepatic, endocrine or renal disease. All subjects were interviewed using the Structured Clinical Interview for DSM-IV Axis I Disorders^42^ to exclude participants with past or current comorbid Axis I diagnoses and to confirm the diagnosis of OCD in the clinical group. As depression is a common comorbidity in OCD, patients also performed the Beck Depression Inventory^41^ and were only included when their score was below 17. In the OCD group, 15 patients were under both antidepressant and antipsychotic treatment, 14 patients were under only antidepressant treatment and 3 patients were medication free. All subjects gave written informed consent to participate in the study. The study was approved by the ethics committee of Izmir Katip Celebi University (No. 119). All methods were carried out in accordance with relevant guidelines and regulations.

OCD severity was rated with the Dimensional Yale–Brown Obsessive–Compulsive Scale (DY-BOCS)^40^. Several studies have indicated that cognitive measures interact with the balance between MF and MB control^18,19,31,32,43^. Thus, we additionally assessed cognitive speed and working memory (Table 1).

### Task

**Figure 4:**
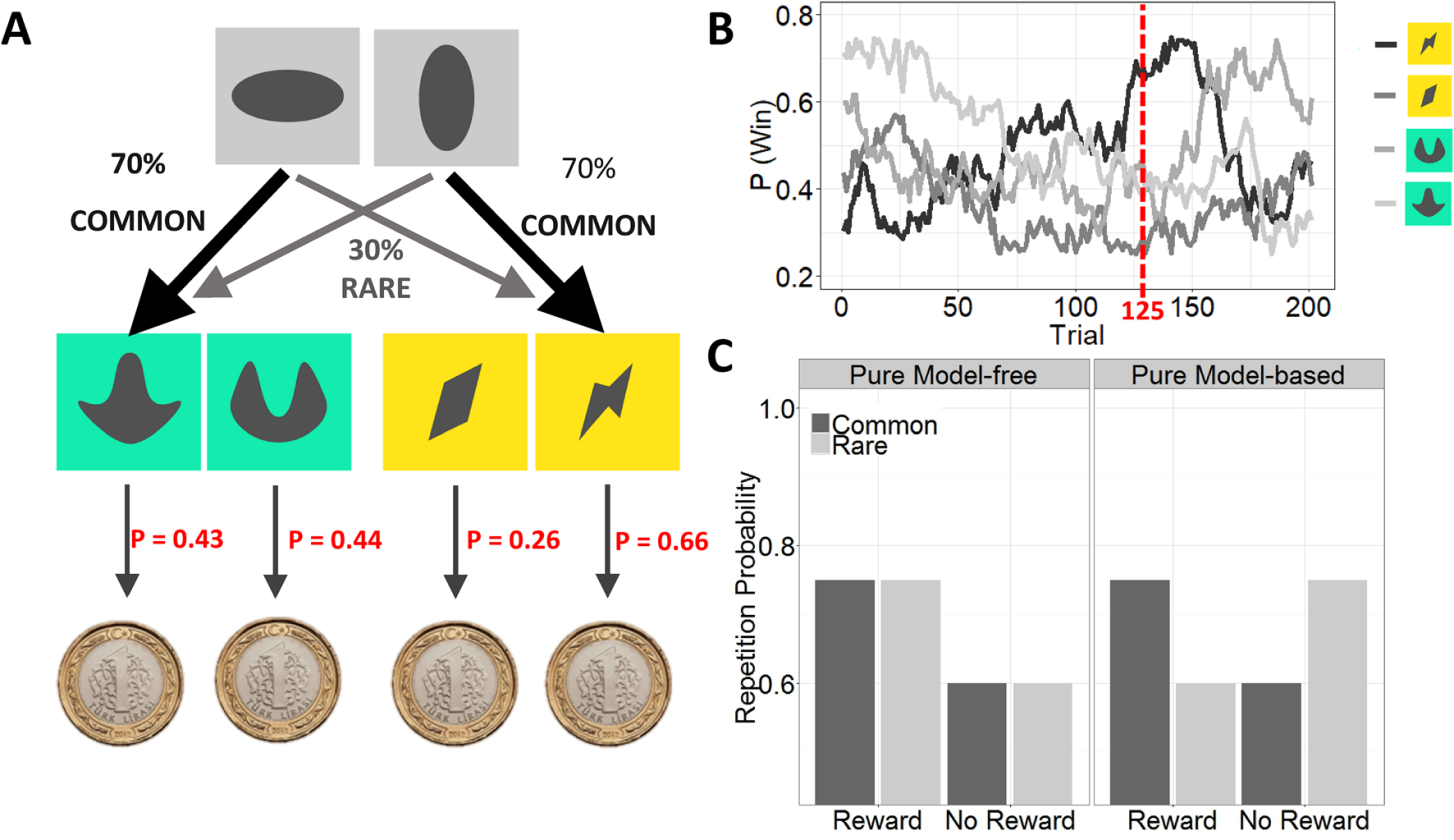
Description of the task. (**A**) One trial of the two-step task. (**B**) Probabilities for 2nd stage outcomes. The win probability for each of the four 2nd stage stimuli varied over time according to a slow and independent random walk (red: example for reward probabilities at trial 125). (**C**) Expected choice behavior for MF vs. MB behavior. The distinction between model-based and model-free performance depends on the use of the transition probability. A purely model-free learner shows a main effect of outcome (Reward vs. No Reward) on 1st stage repetition probability whereas a purely model-based learner shows an interaction between transition frequency (Common vs. Rare) and outcome (Reward vs. No Reward) on 1st stage repetition probability.

As previously^43,44^, we adapted the two-step task for MATLAB with the Psychophysics Toolbox Version 3 extension^45,46^. Participants performed 200 trials of the task. In each trial subjects sequentially performed two choices (Fig. 4A): Each first stage (grey stimuli) choice led to another 2nd stage colored stimulus pair, where subjects again chose one of two stimuli. The probability to be presented with a specific stimulus pair at the 2nd stage depended on the choice at the 1st stage and was constant over time; there was a common (70%) and a rare (30%) transition for each stimulus at the 1st stage. After the 2nd stage choice, subjects received an outcome either Reward (one Turkish lira) or no Reward (turkish lira superimposed by a red cross). The win probability for each of the four 2nd stage stimuli varied over time according to a slow and independent random walk (Fig. 4B). Subjects were instructed to maximize their reward.

The logic of the task is, that model-free and model-based behavior predicts different behavioral patterns at the 1st stage: If choices are model-free, 1st stage decisions that led to a rewarded choice in the previous trial are repeated, independent of whether the previous 1st to 2nd transition was common or rare. Thus model-free behavior predicts a main effect of outcome (Reward vs. no Reward). However, if choices are model-based a decision that led a to a rewarded but rare choice is not repeated, because choosing the alternative 1st stage stimuli is more likely to lead to the rewarding 2nd stage stimulus. Thus model-based behavior incorporates the knowledge of the transition frequencies and thus predicts an interaction between transition frequency and outcome (Fig. 4C).

As explained previously^39^, prior to the task, participants received an instruction that provided detailed information about the structure of the task; specifically concerning the varying outcome probabilities at the 2nd stage and about the constant transition probabilities between the 1st and 2nd stage. In addition, there were 50 practice trials prior to the main experiment. After the experiment, one third of all rewards (with a fixed minimum of 30 and maximum of 60 Turkish Liras) was additionally paid out.

### Statistical analysis of the task

We performed three types of analysis. The first was a mixed effects logistic regression where 1st stage choices (stay/switch) were regressed on the previous trial outcome (Reward/ No Reward), transition frequency (common/ rare) and group (HC, SIB, OCD).

Second, we used a linear mixed effect model to regress 2nd stage RTs on transition frequency (Common, Rare) and group (HC, SIB, OCD) and tested for interactions. This analysis has been suggested to reflect the contribution of the MB system^9,14^, as subjects with stronger representations of the transition frequencies should show stronger discrimination between rare and common trials in their 2nd stage response rates. For further imaging analyses, we performed individual 2nd stage RT effects by substracting individual mean RTs for common trials from rare trials. Note that positive values here relate to larger differences in rare compared to common trials and hence relate to MB control effects.

The third analysis was the fit of the original^3^ reinforcement learning model (7 parameter hybrid model) to the data. This model incorporates model-free and model-based reinforcement learning algorithms (Supplement 7) and weighs between these algorithms by the computational parameter ω. We also fitted alternative models (pure model-free and pure model-based models) to verify that the hybrid model was the best fitting model across all subjects (Supplement 1). We used an expectation maximization algorithm to find maximum a posteriori estimates of the parameters.

As OCD was previously associated with decreased ω parameters from the hybrid model, we tested whether this parameter was lower in OCDs, compared to SIBs, compared to HCs. To this end, we performed nonparametric Jonckheere-Terpstra permutation analysis (10,000 permutations) to test for ordered differences among groups. We performed Post-hoc Wilcoxon rank sum test to indicate which group were significantly different. For the sake of completeness we also compared all other reinforcement learning parameters between groups (Supplement 3).

Regression analysis were conducted using generalized linear mixed-effects models implemented with the lme4 package^47^ in the R programming language, version 3.1.2 (cran.us.r-project.org). Computational modeling was performed in Matlab 2014 (8.3., 2014a). The Jonckheere-Terpstra permutation test was used as implemented in the DescTools package.

### Neuroimaging data

We obtained MRI images using a GE Optima 360 1.5 T scanner (General Electric Medical Systems, Milwaukee, WI, USA). Imaging parameters were: TR = 10.7 ms, TE = 4.3 ms, matrix = 256 × 256, number of slices = 176, FOV = 256 × 256 mm^2^, NEX = 1, slice thickness = 1 mm. The voxels were therefore isotropic with a size of 1 mm^3^. All scans were visually inspected to check for motion artifacts and to rule out gross neuropathology. Structural MRI data was available for 80 subjects (29 OCD, 21 SIB and 30 HC). As described elsewhere^48^, T1 images were analyzed using the FreeSurfer software package (version 6.0, http://surfer.nmr.mgh.harvard.edu). Imaging processing procedures were based on previous reports^49–51^. Subcortical volumes were normalized according to intracranial volumes (ICV) for further statistical analysis.

Cortical thickness and area maps were smoothed with a full width half maximum Gaussian kernel of 10 mm. We performed three sets of correlation analyses as we correlated cortical thickness and surface area with (1) ω values (2) ﻿outcome by transition interaction coefficient and (3) differences in RTs between rare and common trials. Age and gender were added as covariates of no interest. Multiple comparisons were corrected with a Monte Carlo simulation with 10,000 iterations using a threshold of 1.3 (*p* < 0.05). All analyses were performed for the right and left hemispheres separately and p-values were adjusted for two hemispheres.

Previous studies have suggested caudate nucleus and putamen are implicated in MB and MF control systems^5,52^. Therefore, we restricted our analysis of correlation between ω values and normalized subcortical volumes to the bilateral putamen and caudate nucleus with age and gender as covariates of no interest.

## Supplementary Information

Supplementary Information.

## Data Availability

Code and data used in the current study are available from the corresponding author on request.
