# Supplementary material for: Fronto-striatal structures related with model-based control as an endophenotype for obsessive–compulsive disorder
Source: Sci Rep. 2021 Jun 7;11:11951. doi: 10.1038/s41598-021-91179-2 (PMC8185095; doi:10.1038/s41598-021-91179-2)
Supplement: Supplementary file 1 — Supplementary Information. [file 41598_2021_91179_MOESM1_ESM.docx]

**Supplementary Information: Fronto-striatal structures related with model-based control as an endophenotype for obsessive-compulsive disorder**

Meltem I. Kasal^1^, Lutfullah Besiroglu^1^, Nabi Zorlu^1,*^, Nur Dikmeer^1^, Aslıhan Bilge^1^, Ercan Durmaz^1^, Serap Polat^1^, Fazil Gelal^2^, Michael Rapp^3^, Andreas Heinz^4^, Miriam Sebold^3,4^

# Supplementary Information 1: Model selection procedure and model comparison

Following previous study (Sebold *et al.,* 2017)**,** we fitted two alternative models to our choice data: 1) a model-free algorithm SARSA (λ), which only captures a main effect of outcome on first stage choices, and 2) a pure model-based algorithm, which considers the interaction between outcome and transition frequencies, but does not capture a main effect of outcome on first stage choices. The overarching aim of these alternative model fittings was the subsequent model comparison, where we aimed to identify the best fitting algorithm for all groups (OCD patients, first degree relatives = siblings, healthy controls). Therefore, we subjected individual model evidences (integrated likelihoods) for all three models to a Bayesian model selection procedure. Across all three groups, the hybrid model was the best fitting model (Exceedance Probability for Controls = 0.736 , Siblings = 0.599, Patients = 0.669, SI Figure 1, left). Moreover, a non parametric kruskal walis test indicated no group differences with regard to the integrated log likelihoods for the best fitting model (χ= 0.43939, *p* = 0.80, SI Figure 1, right).


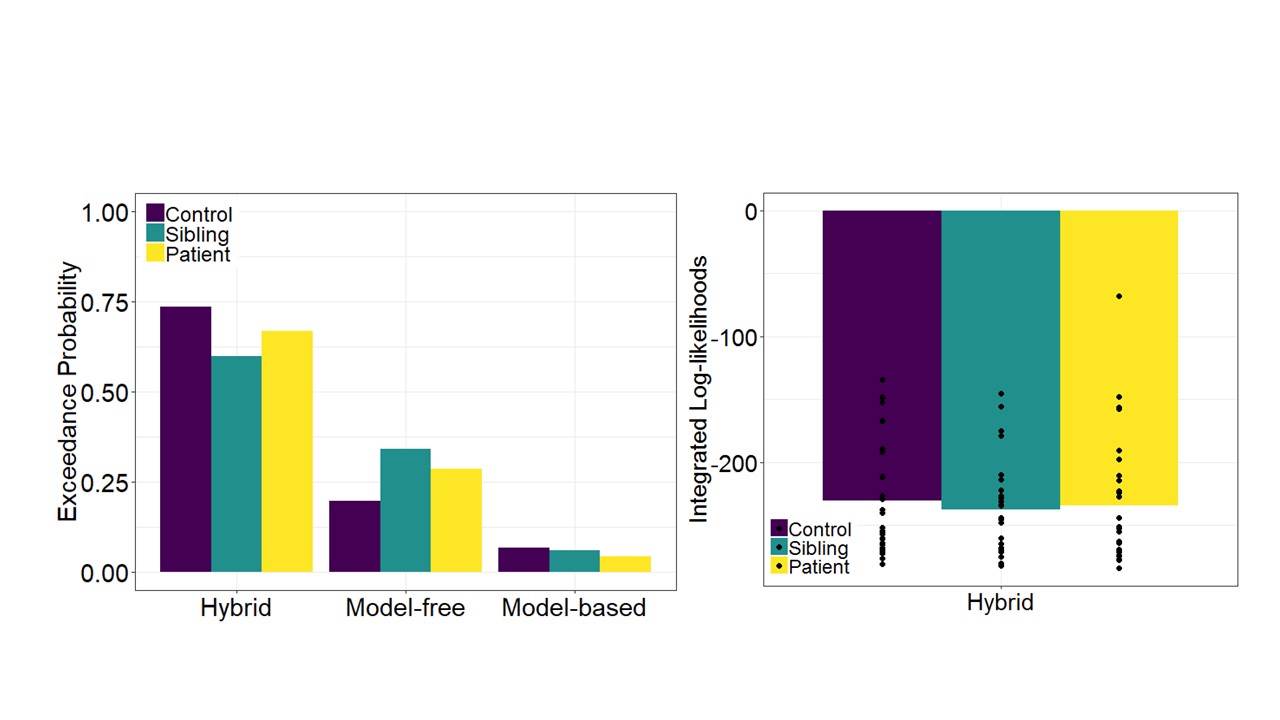


SI Figure 1: Results of Model comparisons depending on group. Left: The hybrid model had the highest exceedance probability and was therefore the best fitting model across all three groups. Right: For the best fitting model (hybrid model) groups did not significantly differ with regard to their integrated log-likelihoods.

# Supplementary Information 2: Association between demographic/ clincial variables and ω values/2nd stage RT effects

Within the patient cohort exploratory analyses were performed to test if the computational parameter ω was associated with clinical variables such as age of onset or OCD severity score (as indicated by YBOCS scores). These analyses (Spearman correlation) revealed no significant associations (ω and age of onset, ρ = -0.238, *p* = 0.187, ω and DYBOCs score, ρ = .002, *p* = .994). Moreover, because we found the strongest between group differences with regard to second stage Reaction times (RT differences between common and rare trials) we also tested associations (spearman correlations) between these model-based indices and both clinical variables within the patient cohort. However, this analyses neither revealed any significant associations (2nd stage RT effects and age of onset; ρ = -0.10, *p* = 0.58, , 2nd stage RT effects and YBOCS score; ρ = -0.060, *p* = .74).

Supplementary Information 3: Effects of Medication Status

Two previous studies found that mediction status in OCD significantly effected model-based control. Whereas (Voon *et al.*, 2015) found that selective serotonine-reuptake inhibitors would decrease reward-related model-based control, a recent study by Gillan *et al.* (2019) showed that medication in general would increase model-based control in OCD and anxiety disorders. Moreover, some studies demonstrated that increasing central dopamine and serotonin levels would increase and decrease model-based control, respectively. (Wunderlich *et al.*, 2012; Worbe *et al.*, 2016; but see Kroemer *et al.*, 2019).

We therefore wanted to test whether mediaction status (antidepressant alone vs. Antidepressant with antipsychotic vs. No mediaction) would impact model-based control, more precisely, the computational parameter ω and the second stage RT effect.

We did not find evidence for the former (Kruskal-walis test ω and Medication status: χ = 3.98, *p* = 0.14). However, we found that second stage RT effects were influenced by medication status (linear mixed model, where we regressed transition and medication status on second stage RTs, with subject as random effect: *p* <.001). Post-hoc analyses indicated that subjects with antidepressant medication showed higher model-based RT effects compared to subjects with antidepressant and antipsychotic medication (*p* = .001, see SI Figure 2) or no medication (p < .01), whereas there was no difference between subjects with combined antidepressant/psychotic vs no medication (*p* =.84)


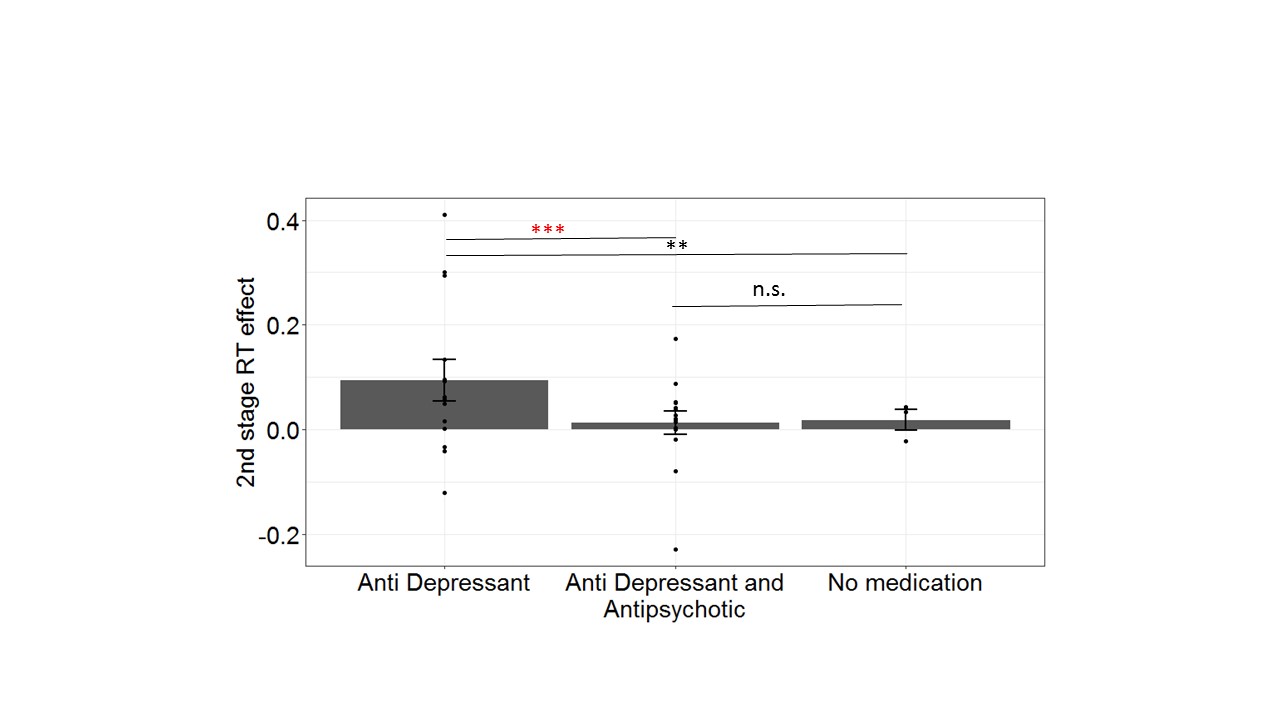


SI Figure 2: 2nd stage RT effects as a function of medication status. Patients with antidepressant medication had significantly higher model-based RT effects compared to patients taking antidepressant and antipsychotic medication.

# Supplementary Information 4: Group comparisons of other model parameters

Neither a non-parametric MANOVA including all model parameters nor an exploratory post-hoc analysis comparing each model parameter seperately between groups (to increase statistical power) resulted in significant between group differences (see SI Table 1)

SI Table 1: Descriptive statistics and result of group analyses for each computational parameter. IQR = inter quartile range

|  | **OCD** | | **SIB** | | **HC** | | **Statistics for group comparison** |
| --- | --- | --- | --- | --- | --- | --- | --- |
| **Parameter** | **median** | **IQR** | **median** | **IQR** | **median** | **IQR** | **Kruskal-Walis Test** |
| **alpha 1** | 0.51 | 0.72 | 0.28 | 0.65 | 0.54 | 0.59 | χ = 2.07, *p* = 0.35, df = 2 |
| **alpha 2** | 0.34 | 0.56 | 0.29 | 0.67 | 0.34 | 0.59 | χ = 0.16, *p* = 0.92, df = 2 |
| **lambda** | 0.44 | 0.86 | 0.35 | 0.64 | 0.72 | 0.67 | χ = 1.78, *p* = 0.41, df = 2 |
| **omega** | 0.09 | 0.41 | 0.37 | 0.51 | 0.50 | 0.66 | χ = 3.34, *p* = 0.19, df = 2 |
| **beta 1** | 3.09 | 6.09 | 3.86 | 9.23 | 3.16 | 5.45 | χ = 0.09, *p* = 0.94, df = 2 |
| **beta 2** | 2.27 | 3.69 | 1.79 | 3.70 | 2.02 | 3.28 | χ = 0.64, *p* = 0.72, df = 2 |
| **repetition** | 0.05 | 0.13 | 0.07 | 0.21 | 0.089 | 0.235 | χ = 1.93, *p* = 0.38, df = 2 |

# Supplementary Information 5: Association between different model-based/ model-free measures

# In order to test how strong different measures of model-based/ model-free control were related to each other we set up a correlation matrix. We found associations between the computational parameter ω, 2nd stage RT effects and the Interaction term but none of these measures were associated with the Outcome term.


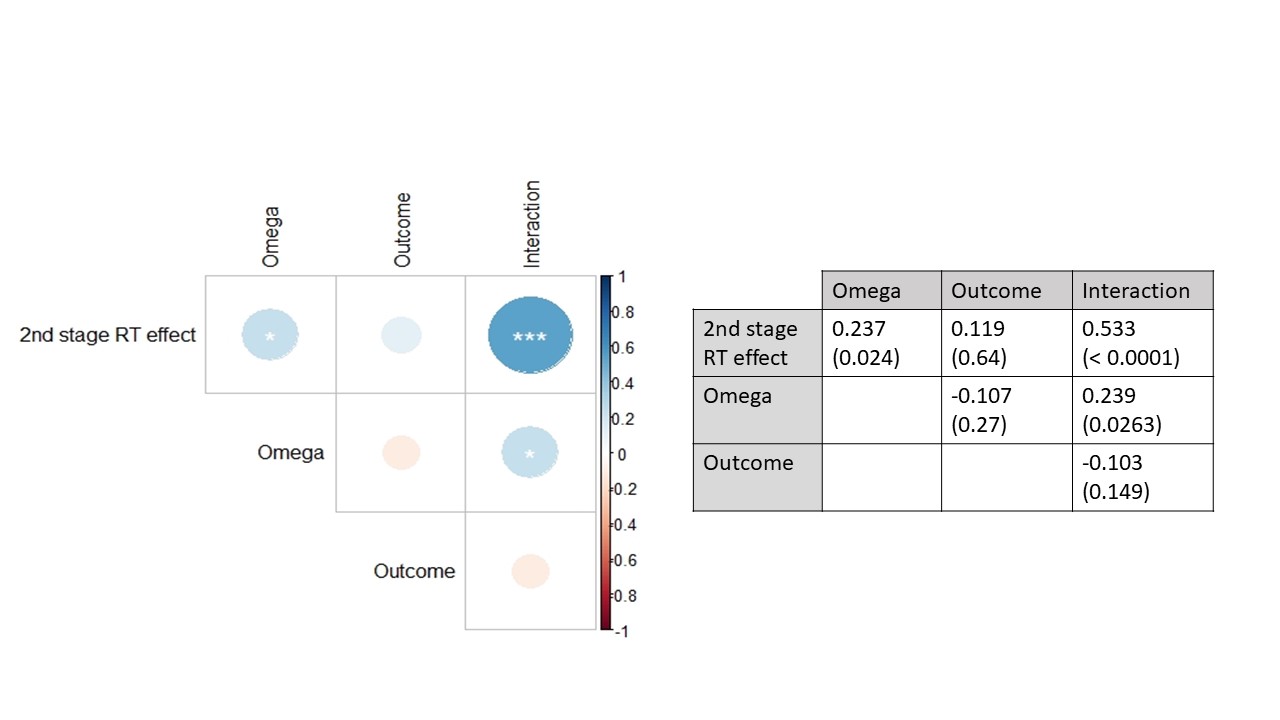


SI Figure 4: Associations between different model-free/ model-based task measures. The table on the right depicts Spearman correlation coefficients (р) and the associated p-values in brackets.

Supplementary Information 6: Correlation between Omega values and cortical thickness and surface area for seperate groups

Exploratory analysis revealed a significant negative correlation between ω values and cortical surface area values of right rostral middle frontal gyrus extending to the pars orbitalis, frontal pole and medial orbitofrontal cortex (Talariach-coordinates x = 35.5, y = 54.4, z = -6.5, size in mm2= 1829.0, CWP = 0.017) within the OCD group, suggesting lower model-based control was related to larger surface area (SI Figure 3A). There were no significant correlations between ω values and cortical thickness and surface area values in the SIB or HC groups.

We found a significant negative correlation between ω values and normalized right putamen for all subjects (ρ = -0.247, *p* = 0.029). There was a significant negative correlation between ω values and normalised right putamen for the HC group (ρ = -0.531, *p* = 0.004), suggesting higher model-based control is related to smaller normalized right putamen volumes (SI Figure 3B). However, none of the correlations reached statistical significance neither in the OCD group (ρ = -0.303, *p* = 0.124) nor in the SIB group (ρ = 0.083, *p* = 0.735). Within all subjects, there was a trendwise significant negative correlation between ω values and normalized left putamen (ρ = -0.196, *p* = 0.086). Follow-up analysis revealed a significant negative correlation between ω values and normalised left putamen for HC group (ρ = -0.530, *p* = 0.004) (SI Figure 3B). However, none of the correlations reached statistical significance in the OCD (ρ = -0.172, *p* = 0.392) or in the SIB groups (ρ = 0.089, *p* = 0.712). We did not find any significant correlations between ω values and normalised bilateral caudate nucleus values within each subjects.


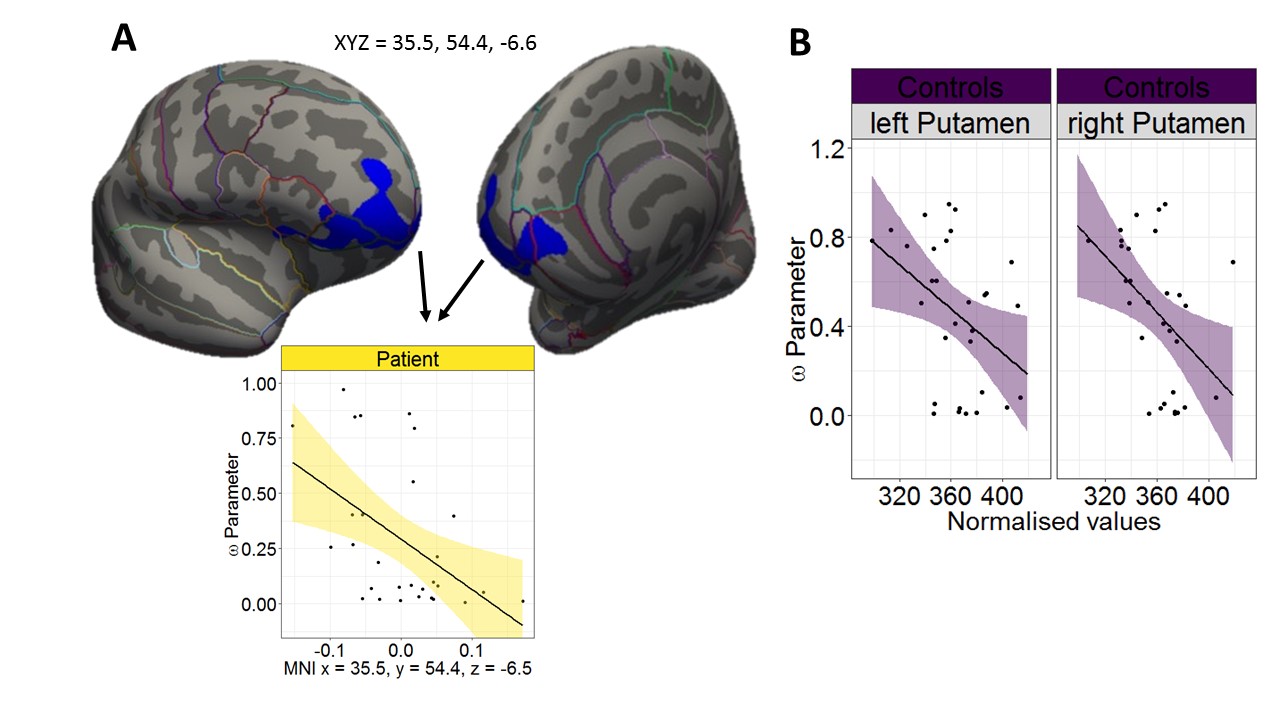


SI Figure 3: Association between Omega values and neuroimaging findings. A.) We found a significant negative correlation between Omega values and surface area in the middle frontal gyrus in patients only. We found a significant negative association between ω values and normalised right and left putamen in the healthy controls only

# Supplementary Information 7: Computational Modelling

The algorithm of the 7-parameter hybrid model includes both model-based and model-free components, which allows for mapping state-action pairs to expected future values.

The model-free strategy is computed using the SARSA (λ) temporal difference learning. At each stage *i* of each trial *t,* the value for each state-action pair was calculated as follows:

*Q_TD_ (s_i,t_,a_i,t_) = Q_TD_ (s_i,t_,a_i,t_) + α_i_ δ_i,t_*

where *δ_i,t_ = r_i,t_ + Q_TD_ (s_i+1,t_ , a_i + 1,t_) - Q_TD_ (s_i,t_ ,a_i,t_)* and *α_i_*  is a free learning parameter. Different learning rates *α_1_* and *α_2_* for the two task stages were estimated. The reinforcement eligibility parameter (λ) determines the update of the first-stage action by the second-stage prediction error as follows:

*Q_TD_ (s_1,t_ , a_i1,t_) = Q_TD_ (s_1,t_ ,a_1,t_) + a_1_λδ_2,t ._*

The model-based reinforcement-learning algorithm was computed by mapping state-action pairs to a transition function and assuming that participants choose between two possibilities, as follows:

*P(S_B_ ⏐S_A_, a_A_) = 0.7, P(S_C_ ⏐S_A_, a_B_) = 0.7* for common

and

*P(S_B_ ⏐S_A_, a_A_) = 0.3 P(S_C_ ⏐S_A_, a_B_) = 0.3* for rare transitions,

where S is the state (first stage: S_A_; second stage: S_B_ and S_C_), and a is the action (two actions: a_A_ and a_B_) at a given state. The action value (Q_MB_) was computed at each trial from the estimates of the transition probabilities and outcomes and was defined for the first stage as follows:

*Q_MB_ (s_A_,a_i_) = P(s_B_|s_A_,a_i,_) max_a_ Q_TD_(s_B_,a) + P(s_C_|s_A_,a_i,_) max_a_ Q_TD_(s_C_,a)*

Finally, to connect values to choices, the weighted sum of the model-free and model-based values was computed for the first stage as defined:

*Q_net_(s_A_,a_j_) = w Q_MB_(s_A_,a_j_) + (1-w) Q_TD_(s_A_,a_j_)*

where *w* is the weighting parameter. Assuming that two approaches coincide at the second stage, and that Q_MB_ = Q_TD_, at the second stage Q_net_= Q_MB_ = Q_TD._ Then, the probability of a choice is the softmax equation for

Q_net_:

*Σ_a’_ exp (β_i_[Q_net_(s_i,t_,a) + p * rep(a)])*

*P(a_i,t_ = a|s_i,t_) = exp (β_i_[Q_net_(s_i,t_,a) + p * rep(a)]) /*

where the free inverse temperature parameters (β_i_) control the choice randomness, and *p* captures perseveration (p > 0) or switching (p < 0) in the first-stage choices. In total, the hybrid model contains 7 free parameters (β1, β2, α1, α2, λ, p, ω), with special cases of pure model-based (ω = 1) and model-free (ω = 0) models.

References:

Gillan, C.M. *et al.* Comparison of the Association Between Goal-Directed Planning and Self-reported Compulsivity vs Obsessive-Compulsive Disorder Diagnosis. *JAMA Psychiatry* **77**, 1-10 (2019).

Kroemer, N.B., Lee, Y., Pooseh, S., Eppinger, B., Goschke, T., Smolka, M.N. L-DOPA reduces model-free control of behavior by attenuating the transfer of value to action. *Neuroimage* **186,** 113-125 (2019).

Sebold, M. *et al*. When Habits Are Dangerous: Alcohol Expectancies and Habitual Decision Making Predict Relapse in Alcohol Dependence. *Biological Psychiatry* **82**, 847–856 (2017).

Voon, V. *et al.* Motivation and value influences in the relative balance of goal-directed and habitual behaviours in obsessive-compulsive disorder. *Translational psychiatry* **5,** e670 (2015).

Worbe, Y *et al.* Valence-dependent influence of serotonin depletion on model-based choice strategy. *Molecular Psychiatry* **21**, 624-629 (2016).

Wunderlich, K., Smittenaar, P., Dolan, R.J. Dopamine enhances model-based over model-free choice behavior. *Neuron* **75**, 418-424 (2012).
